# Functional characterization of dopamine transporter *in vivo* using *Drosophila melanogaster* behavioral assays

**DOI:** 10.3389/fnbeh.2014.00303

**Published:** 2014-09-03

**Authors:** Taro Ueno, Kazuhiko Kume

**Affiliations:** ^1^Department of Stem Cell Biology, Institute of Molecular Embryology and Genetics, Kumamoto UniversityKumamoto, Japan; ^2^Department of Sensory and Motor Systems, Tokyo Metropolitan Institute of Medical ScienceSetagaya, Tokyo, Japan; ^3^Department of Neuropharmacology, Graduate School of Pharmaceutical Sciences, Nagoya City UniversityMizuho, Nagoya, Japan

**Keywords:** sleep, dopamine, dopamine transporter, memory, *Drosophila melanogaster*, volume transmission

## Abstract

Dopamine mediates diverse functions such as motivation, reward, attention, learning/memory and sleep/arousal. Recent studies using model organisms including the fruit fly, have elucidated various physiological functions of dopamine, and identified specific neural circuits for these functions. Flies with mutations in the *Drosophila* dopamine transporter (*dDAT*) gene show enhanced dopamine signaling, and short sleep and memory impairment phenotypes. However, understanding the mechanism by which dopamine signaling causes these phenotypes requires an understanding of the dynamics of dopamine release. Here we report the effects of *dDAT* expression on behavioral traits. We show that *dDAT* expression in a subset of dopaminergic neurons is sufficient for normal sleep. *dDAT* expression in other cell types such as Kenyon cells and glial cells can also rescue the short sleep phenotype of *dDAT* mutants. *dDAT* mutants also show a down-regulation of the D1-like dopamine receptor *dDA1*, and this phenotype is rescued when* dDAT* is expressed in the same cell types in which it rescues sleep. On the other hand, *dDAT* overexpression in mushroom bodies, which are the target of memory forming dopamine neurons, abolishes olfactory aversive memory. Our data demonstrate that expression of extrasynaptic dopamine transporters can rescue some aspects of dopamine signaling in dopamine transporter mutants. These results provide novel insights into regulatory systems that modulate dopamine signaling.

## Introduction

The neurotransmitter dopamine is crucial for arousal, motor function and memory. Further, accurate temporal and spatial regulation of dopamine signaling is required for these activities. The dopamine transporter (*DAT*) mediates reuptake of dopamine from the synaptic cleft, and has a key role in limiting dopamine signaling. DAT is inhibited by therapeutic agents such as Ritalin (methylphenidate) or psychostimulant drugs of abuse such as amphetamine and cocaine, demonstrating its importance in regulating behaviors. Besides directly regulating dopamine signaling, *DAT* is thought to participate in gating the spillover of dopamine (Floresco et al., 2003; Cragg and Rice, 2004). This spillover of dopamine from a release site into the extrasynaptic space (i.e., volume transmission) has been reported in mammals, and is proposed to modulate brain function.

The *Drosophila dDAT* mutant, *fumin (fmn)*, sleeps less than wild-type flies due to an increase in postsynaptic dopamine signaling (Kume et al., 2005). *In vivo* voltametric studies have shown that the *fumin* mutants are defective for dopamine clearance (Makos et al., 2009). Increased dopamine signaling in *fumin* mutants also alters their activity, metabolic rate, longevity, temperature sensitivity and sensitivity to dietary calories (Ueno et al., 2012a,b; Yamazaki et al., 2012). A similar effect is seen in mammals, where deletion of the dopamine transporter gene results in a decrease in sleep (Wisor et al., 2001). Recent studies have identified a dopamine pathway for controlling fly sleep (Liu et al., 2012b; Ueno et al., 2012c) which is distinct from other dopamine pathways responsible for memory formation (Aso et al., 2010), and the response to ethanol (Kong et al., 2010).

Due to its powerful genetics, *Drosophila* is an ideal model organism for identifying genes or neural circuits underlying specific behaviors. Currently, little is known about the dynamics and distribution of the released dopamine. For example, it is unknown how far dopamine, released from specific sites, diffuses, and how long the effects of released dopamine last. To investigate the effects of released neurotransmitters, genetic perturbation of plasma membrane transporters can be used to great advantage for controlling the intensity of extracellular signals.

In this report, we describe effects of altered *dDAT* expression on fly behavior. Using tissue specific knockdown and rescue experiments, we found that *dDAT* expression in a subset of dopaminergic neurons is sufficient for normal sleep. In addition, we also found that *dDAT* expression in glial cells could rescue the short sleep phenotype of *fumin* mutants. *dDAT* expression in postsynaptic sites for memory formation (the mushroom bodies), abolished olfactory memory formation. These results shed light on the physiological function of *dDAT* in dopamine signaling and in regulating extrasynaptic dopamine spillover *in vivo*.

## Materials and methods

### Fly stocks

Flies were reared on a standard corn meal, yeast, glucose agar medium at 24.5°C under a 12 h:12 h light:dark cycle. The *fmn* (*fumin*) mutant was described previously (Kume et al., 2005). TH-GAL4 was a gift from J. Hirsh. 104Y was a gift from D. Armstrong. NP lines were obtained from the Kyoto *Drosophila* Genetic Resource Center, To remove possible modifiers and allow comparisons in a common genetic background, we outcrossed all the alleles into the *w*^1118^ or *fmn* background over at least five consecutive generations. As controls we used *w*^1118^ or *fmn* flies. The transgenic RNA interference (RNAi) line for *DAT* (Transformant ID: 106961), *UAS*-*Dicer*-*2* flies (Stock numbers: 60008) and the *w*^1118^ (60000) which is the genetic background of the RNAi line were obtained from the Vienna *Drosophila* RNAi Center (VDRC), Vienna, Austria (Dietzl et al., 2007).

### Sleep analysis

Flies were placed individually in glass tubes (length, 65 mm; inside diameter, 3 mm) containing 1% agar and 5% sucrose at 24.5°C. Male flies were used for sleep analysis. They were entrained for at least 3 d to LD conditions before being transferred to constant dark (DD) conditions. Locomotor activity was monitored by recording infrared beam crossings by individual flies in 1 min bins using the *Drosophila* activity monitoring system (Trikinetics, Waltham, MA, USA). Based on previous reports (Hendricks et al., 2000; Shaw et al., 2000), sleep was defined as periods of inactivity lasting 5 min or longer. For the pharmacological manipulations, 3-iodo-d-tyrosine and RU486 were obtained from Sigma-Aldrich. Flies were transferred to vehicle (1% agar + 5% sucrose) with 3 mM 3-iodo-d-tyrosine, 0.5 mM RU486. Daily sleep time was calculated with software written in Excel or R 2.11.1.

### Measurements of transcript levels

The efficiency of *dDAT* RNAi were examined by qPCR. Total RNA was extracted from 10 heads by using RNeasy Micro Kit (Qiagen) according to the manufacturer’s instructions. cDNA was synthesized from the total RNA using oligo (dT)_20_ primer and ReverTra Ace reverse transcriptase (Toyobo). The cDNA was used for qPCR using THUNDERBIRD SYBR qPCR Mix (Toyobo). The primers were 5′-AACAATAGCA TCAGCGACGA-3′ and 5′- CAGGTTATGG CACCCTTACG-3′ for *dDAT*, 5′- TGGTACGACA ACGAGTTTGG-3′ and 5′- TTTCAGGCCG TTTCTGAAGT-3′ for *GAPDH2*. Before qPCR, all the primer sets were confirmed to yield one major band corresponding to each targeted mRNA on the gel when used in conventional RT-PCR. Each expression level was first normalized to *GAPDH2*. Then, the values were normalized to the average of independent control sample.

### Immunohistochemistry

Brains of adult flies were dissected in cold PBS and fixed in 4% paraformaldehyde in PBS for 20 min at room temperature. After three 20 min washes in 0.3% Triton X-100 in PBS, samples were blocked and penetrated in 5% normal sheep serum (NSS) for 30 min at room temperature. Samples were then incubated in a primary antibody solution in 5% NSS at 4°C overnight. The following primary antibodies were used in this study: chicken anti-GFP (1:1000; Abcam), rabbit anti-TH (1:25 Pel-Freez), rat anti-DAT (1:1000; Millipore) and rabbit anti-dDA1 (1:1250: gift from Dr. Fred W. Wolf). After three 20 min washes in 0.3% Triton X-100 in PBS, brains were incubated at 4°C overnight in a secondary-antibody solution in 5% NSS. The following secondary antibodies were used: Alexa Fluor 568 goat anti-rat IgG (1:200; Invitrogen), Alexa Fluor 488 goat anti-chicken IgG (1:200; Invitrogen) and Alexa Fluor 568 goat anti-rabbit IgG (1:200; Invitrogen). After three 20 min washes in PBS, brains were mounted using PermaFluor (Thermo Scientific) between two coverslips separated by electrical tape of ~200 μm thickness, so that the brain sample was not flattened. Immunolabeled adult brains were imaged under a Leica TCS SP2 AOBS confocal microscope using 20x magnification. Z-stack images were scanned at 2 μm section intervals with a resolution of 1024 × 1024 pixels.

For quantification of dDA1 signal, a confocal slice image containing the holizontal lobes of the mushroom body was selected from a stack. The average pixel intensity values in the horizontal lobes and the adjacent control region (aimpr: anterior inferior medial protocerebrum) was measured by NIH ImageJ. The signal of the dDA1 in horizontal lobes was normalized by the control region of an identical confocal slice.

### Aversive odor memory

Olfactory conditioning with two odors (4-methylcyclohexanol and 3-octanol) was performed as described previously (Tully and Quinn, 1985). Briefly, approximately 100 flies were placed in a training chamber where they were exposed to odors and electric shocks. One of the aversive odors, OCT (3-octanol, Sigma) or MCH (4-methylcyclohexanol, Sigma), was paired with 12 pulses of electrical shocks (60 V DC) for 1 min, whereas the other was not. Testing was performed for 2 min after the training by placing flies at a choice point in a T-maze between the two odors for 2 min. If no flies moved to either arm of the T-maze or if a small number of flies were trapped in the middle compartment, it indicated that the flies did not choose either odor. A performance index (PI) was calculated so that a 50:50 distribution (no memory) yielded a PI of 0 and a 0:100 distribution away from the shock-paired odor yielded a PI of 100. Individual PIs were always the average of two experiments where the shock-paired odor was alternated.

### Statistical analysis

The significance level in each experiment was set to 5%. Comparisons were made by using either Student’s *t*-test (for two groups) or analysis of one-way ANOVA with Tukey-Kramer HSD *post hoc* test (for more than two groups). Bars and error bars represent means and SEM, respectively. Data were analyzed as described in the figure legends using R 2.11.1.

## Results

### Short sleep phenotype in *fumin* and *dDAT* knockdown flies

**Figure 1:**
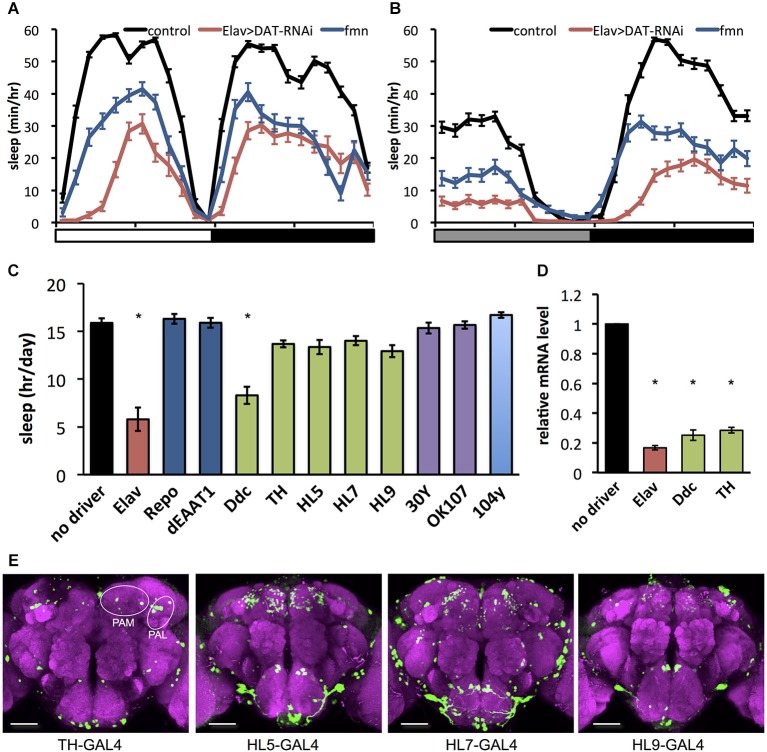
**Effect of** ***dDAT* knockdown on sleep. (A)** Sleep profiles in 60 min intervals for each genotype (*n* = 32 flies). Sleep was monitored in 12 h light: 12 h dark condition (LD). Day and night are depicted by the white and black bars, respectively. **(B)** Sleep profiles in 60 min intervals for each genotype (*n* = 16–32 flies). Sleep was monitored in constant darkness condition (DD). Subjective day and night are depicted by the gray and black bars, respectively. **(C)** Total daily sleep amounts for flies with *dDAT* knockdown using various GAL4 drivers (*n* = 16–32 flies). Data are represented as the mean ± s.e.m. * *P* < 0.05; one-way ANOVA with Tukey-Kramer HSD *post hoc* test. **(D)** Efficiency of *dDAT* knockdown. The expression levels of *dDAT* gene in the head of flies expressing *dDAT* RNAi transgene by GAL4 drivers are expressed as the relative values to the control flies. *n* = 3 for each group. Data are represented as the mean ± s.e.m. * *P* < 0.05; one-way ANOVA with Tukey-Kramer HSD *post hoc* test. **(E)** Expression patterns of GAL4 drivers for dopaminergic neurons in the central brain. The GAL4 expression for each driver was visualized using UAS-mCD8::GFP (green). The brain was stained with antibody to nc82 to stain the neuropil (magenta). PAM, protocerebral anterior medial; PAL, protocerebral anterior lateral. Scale bars = 50 μm.

As previously reported (Kume et al., 2005), the *dDAT* mutant *fumin* shows a short sleep phenotype (Figures 1A,B). To further investigate behavioral effects of *dDAT* expression, we examined the sleep phenotype of *dDAT* knockdown flies. Using GAL4/UAS system (Brand and Perrimon, 1993), we expressed dsRNA to induce RNAi in a cell specific manner. As shown in Figures 1A,B, flies with pan-neuronal knockdown of *dDAT* using Elav-GAL4 displayed a significant sleep reduction compared with control flies. The total daily sleep of *dDAT* RNAi flies decreased to about half that of control flies (Figure 1C). Next, we examined the effect of *dDAT* RNAi in specific cells or various brain regions using a series of GAL4 drivers. We performed this GAL4 driver screening in constant darkness since light has buffering effect against wake promoting effect of dopamine (Shang et al., 2011). It has been reported that *dDAT* gene expression is restricted to dopaminergic neurons (Porzgen et al., 2001). Consistent with the previous reports, *dDAT* knockdown in glial cells resulted in no significant sleep decrease. Flies subjected to *dDAT* knockdown in the dopamine neurons using Ddc-GAL4 showed a significantly short sleep time compared with control. On the other hand, *dDAT* knockdown in dopaminergic neurons using TH-GAL4 resulted in no significant sleep reduction. Ddc-GAL4 labels dopamine neurons including PAM cluster neurons, whereas this cluster neurons are sparcely labeled by the TH-GAL4 (Liu et al., 2012a). To check the efficiency of RNAi, we quantified *dDAT* mRNA level using qPCR. As shown in Figure 1D, pan-neuronal knockdown of *dDAT* using Elav-GAL4 decreased *dDAT* mRNA level to one-fifth of control. *dDAT* knockdown in dopaminergic neurons using TH-GAL4 or Ddc-GAL4 both showed decreased *dDAT* mRNA level to one-fourth of control, which is consistent with the previous report that showed *dDAT* expression is restricted to dopaminergic neurons. These results suggest that GAL4 expression pattern rather than GAL4 expression level contribute to the sleep phenotype. To further investigate the effect of *dDAT* knockdown in a subset of dopaminergic neurons, we crossed with HL5-, HL7-, HL9-GAL4 drivers. These drivers have GAL4 expression in PAM clusters whereas other clusters such as PPL1 neurons are sparcely labeled (Claridge-Chang et al., 2009; Figure 1E). Despite dense expression in PAM clusters, *dDAT* knockdown using these drivers showed no significant sleep reduction. We further knocked down *dDAT* in mushroom body and fan-shaped body. These neuropiles receive projection of dopaminergic neurons and contribute to arousal effect of dopamine (Andretic et al., 2008; Liu et al., 2012b; Ueno et al., 2012c). Consistent with Portzgen et al. that showed restricted *dDAT* expression in dopamine neurons, *dDAT* knockdown in these postsynaptic neurons has no effect on sleep phenotype. Taken these results together, we hypothesize that *dDAT* expression in a subset of dopamine neurons is sufficient for the reuptake of dopamine from synaptic cleft then shut down postsynaptic dopamine signaling for normal sleep.

### *dDAT* expression in a subset of dopamine neurons can rescue the short sleep phenotype in *fumin* mutants

**Figure 2:**
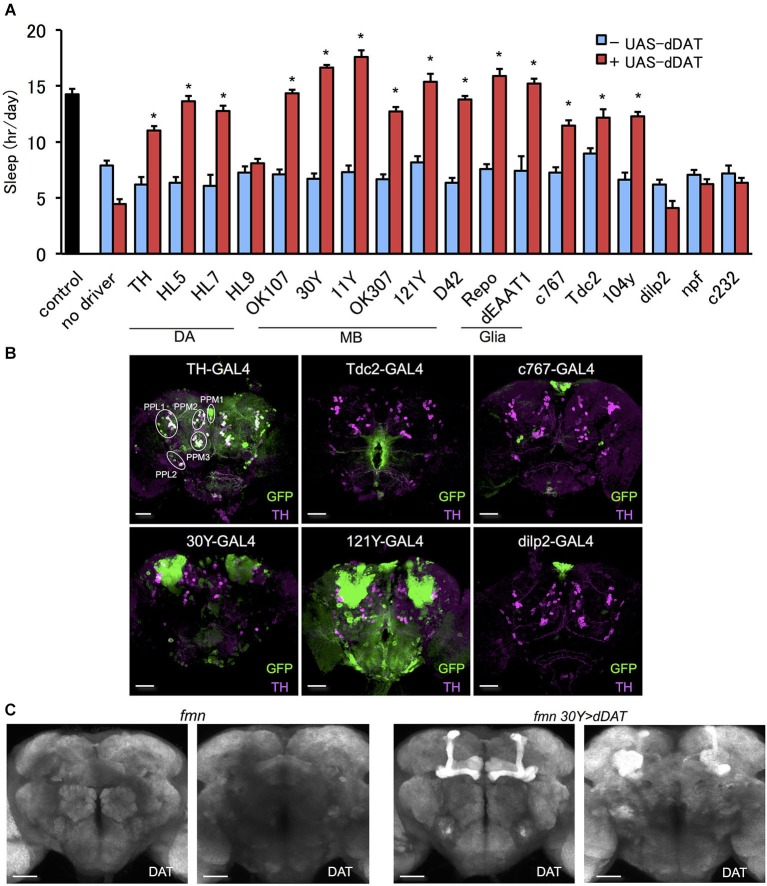
**Effect of *dDAT* expression on sleep. (A)** The short sleep phenotype of *fmn* was rescued by expression of *dDAT* in various brain regions. Black bar indicates control fly without *dDAT* mutation. Blue bars indicate *fmn* GAL4 drivers without *UAS-dDAT*. Red bars indicate *fmn* GAL4 drivers with *UAS-dDAT* (*n* = 16–32 flies). Error bars represent s.e.m. * *P* < 0.05; two-sided Student’s *t*-test. **(B)** Expression patterns of GAL4 drivers in the central brain. The GAL4 expression for each driver was visualized using UAS-mCD8::GFP (green). Dopamine neurons were visualized using anti-TH antibody counterstaining (magenta). PPM, protocerebral posterior medial; PPL, protocerebral posterior lateral. Scale bars = 50 μm. **(C)** *dDAT* expression in the fly brain detected with immunohistochemistry. *dDAT* expression in *fmn* flies and *fmn 30Y > dDAT* flies are shown. Left panel shows anterior view and right panel shows posterior view. Scale bars = 50 μm.

To further investigate the effect of *dDAT* expression on fly sleep, we rescued *dDAT* in *fumin* mutant. Using GAL4 drivers which labels a subset of dopamine neurons, we expressed *dDAT* in dopaminergic neurons in *fumin* mutant. Although we could not observe significant sleep reduction with *dDAT* knockdown using TH-GAL4 (Figure 1C), *fumin* with *dDAT* expression in TH-GAL4 labeled neurons showed significant increase of sleep (Figure 2A). As described above, dopaminergic neurons except for PAM cluster neurons are labeled by TH-GAL4 line. Other GAL4 drivers which labels a subset of dopaminergic neurons, HL5- and HL7-GAL4, could also rescue the short sleep phenotype of *fumin* by cell specific *dDAT* expression. HL5-GAL4 has dense expression in PAM cluster neurons whereas PPL1 and PPM3 cluster neurons which are one of the wake promoting dopaminergic neurons are not labeled by this GAL4 driver. HL7-GAL4 also labeles PAM cluster neurons but PPL1 and PPM3 clusters are sparcely labeled (Kong et al., 2010). Based on the discrepancy between *dDAT* knockdown and *dDAT* rescue experiments, we consider that dDAT expression in a subset of dopamine neurons is sufficient to rescue the short sleep phenotype of *fumin* mutant. On the other hand, *fumin* with *dDAT* expression by HL9-GAL4 showed no significant increase of sleep. It might be due to its low expression level since we observed lower expression in HL9-GAL4 compared with HL5- and HL7-GAL4 drivers.

### *dDAT* expression in non-dopaminergic cells can rescue the short sleep phenotype in *fumin* mutants

Since we could rescue the short sleep phenotype of *fumin* by *dDAT* expression in different subset of dopaminergic neurons which were insensitive to *dDAT* knockdown, we further investigated the effect of ectopic dDAT expression in non-dopaminergic cells. We presumed if *dDAT* expression in a subset of dopamine neurons is sufficient for the shut down of postsynaptic dopamine signaling, it is possible to control dopamine signaling by *dDAT* expression in postsynaptic neurons or extrasynaptic cells such as glia.

In order to express *dDAT* in various regions of the brain in a *fumin* background, we outcrossed the GAL4 drivers to the *fumin* mutant. Next, we examined the effect of *dDAT* expression on the short sleep phenotype of the *fmn* mutant. We found that *fmn* flies with *dDAT* expression in non-dopaminergic neurons showed a significant sleep increase (Figure 2A). Mushroom body (Joiner et al., 2006; Pitman et al., 2006) and fan-shaped body (Donlea et al., 2011) receive dopaminergic projection and regulates sleep and arousal. Although we observed no significant changes in sleep by *dDAT* knockdown in these postsynaptic structure (Figure 1C), *dDAT* expression in these neuropile rescued short sleep of *fumin* mutant. In addition, *dDAT* expression in glial cells also rescued short sleep in *fumin*. To confirm that the drivers have no GAL4 expression in dopamine neurons, we expressed GFP using the GAL4/UAS system and then counter stained with anti-TH antibody. As the merged signal patterns shown in Figure 2B, TH-GAL4 drives GFP expression in most, but not all, dopamine neurons. Conversely, we could not detect any merged signals when expressing GFP using the other drivers, which suggests that these drivers only expressed *dDAT* in non-dopaminergic neurons. Next, we analyzed the localization of ectopically expressed *dDAT* using immunohistochemistry. Although we could not detect endogeneous *dDAT* expression due to high background with the antibody, ectopic expression of *dDAT* in the *fmn* mutant using the 30Y-GAL4 driver, which has strong expression in the mushroom body, showed *dDAT* immunostaining in all lobes and calyx of the mushroom body (Figure 2C). These lobes and calyx are composed of axon bundles and dendrites of Kenyon cells, thus ectopically expressed *dDAT* appears to be transported and localized to the synaptic area.

**Figure 3:**
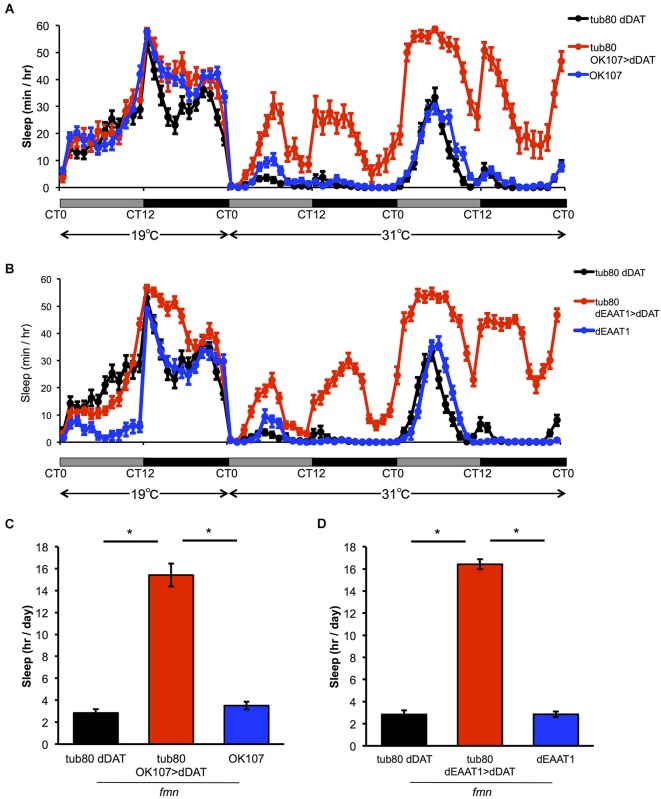
**Effect of spatiotemporal *dDAT* expression on sleep. (A)** Sleep profiles and daily sleep amounts for flies expressing *dDAT* in the mushroom body using the TARGET system.** (B)** Sleep profiles and daily sleep amounts for flies expressing *dDAT* in glial cells using the TARGET system. **(C)** Total daily sleep amounts for flies expressing *dDAT* in the mushroom body using the TARGET system. Daily sleep at 31°C is shown. **(D)** Total daily sleep amounts for flies expressing *dDAT* in glial cells using the TARGET system. Daily sleep at 31°C is shown. Data are represented as the mean ± s.e.m. * *P* < 0.05; one-way ANOVA with Tukey-Kramer HSD *post hoc* test (*n* = 32 flies).

To further investigate if the effect of dDAT expression in non-dopaminergic neurons is due to developmental changes or not, we expressed dDAT in postsynaptic neurons or glial cells using spatiotemporal expression system, TARGET system (McGuire et al., 2003). In this system, dDAT expression is suppressed at 19°C by the temperature sensitive GAL4 inhibitor GAL80^ts^ which is under the control of *tubulin* promotor. After shifting to 31°C, GAL80^ts^ was inactivated and *dDAT* expression will be induced. All the flies were raised at 19°C to adulthood. At the permissive temperature, progeny from the experimental crosses showed similar sleep patterns to control flies. The temperature shift to 31°C significantly increased sleep time of flies which has temporal *dDAT* expression in mushroom body and glial cells (Figure 3). These results suggest that *dDAT* expression in non-dopaminergic neurons, such as the mushroom body or glial cells, can rescue the short sleep phenotype in *fmn* without developmental changes.

### Enhancement of extracellular dopamine causes downregulation of the D1-like receptor *dDA1*

**Figure 4:**
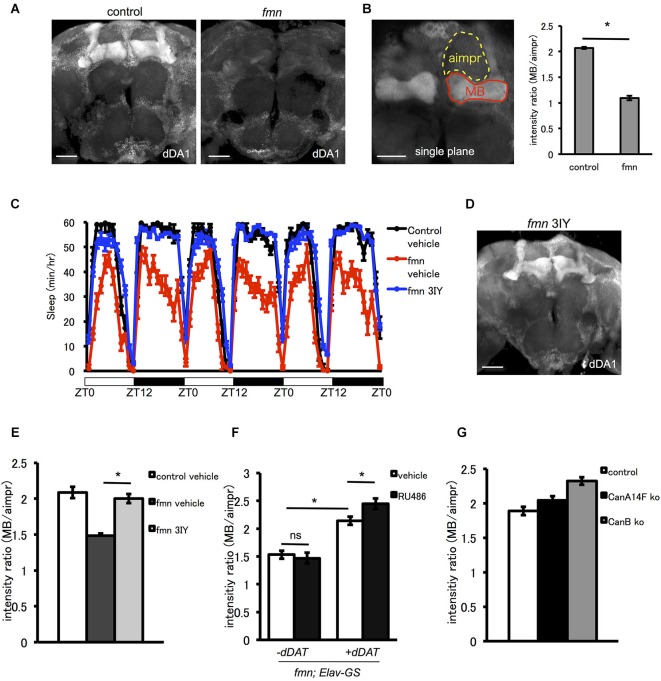
**The D1-like receptor** ***dDA1* in the mushroom body is regulated by dopamine signaling. (A)** *dDA1* expression in the mushroom body. *dDA1* expression in the mushroom body was visualized using the anti-*dDA1* antibody. Robust *dDA1* expression in the mushroom body was detected in the control, on the other hand, the *fmn* mutant showed decreased *dDA1* expression in the mushroom body. Images using the maximum projection are shown. Scale bars = 50 μm. **(B)** Quantification of *dDA1* expression. *dDA1* expression was quantified by calculating the signal ratio of the horizontal lobe and the aimpr. The *fmn* mutant shows significantly decreased *dDA1* expression compared with the control. * *P* < 0.05; two-sided Student’s *t*-test. Scale bar = 50 μm. **(C)** The dopamine synthesis inhibitor 3IY rescues the short sleep phenotype of *fmn* mutants. *fmn* flies fed with vehicle (red line) show decreased sleep compared with control flies (black line), on the other hand, *fmn* flies fed with 3IY (blue line) show sleep levels similar to the control (*n* = 16 flies). **(D)** *dDA1* expression was restored in *fmn* mutants fed with 3IY. *dDA1* expression in the mushroom body was visualized using the anti-*dDA1* antibody. Image using the maximum projection is shown. Scale bar = 50 μm. **(E)** Quantification of *dDA1* expression in the mushroom body with drug administration. *dDA1* expression in the mushroom body was calculated as shown in Figure 4B. * *P* < 0.05; two-sided Student’s *t*-test. **(F)** Quantification of *dDA1* expression in the mushroom body with temporal *dDAT* expression. *dDAT* expression was induced using the Elav-GeneSwitch. *dDA1* expression of flies with or without RU486 0.5 mM administration for each genotype are shown. * *P* < 0.05; one-way ANOVA with Tukey-Kramer HSD *post hoc* test. **(G)** Quantification of dDA1 expression in the short sleep mutant. *dDA1* expression of flies with either the *CanA14F* knockout or the *CanB* knockout are shown.

The D1-like receptor *dDA1* is a dopamine receptor expressed in the mushroom body and in central complex structures such as the ellipsoid body and fan-shaped body. *dDA1* expression in the mushroom body mediates olfactory memory (Kim et al., 2007). As shown in Figure 4A, *fmn* mutants demonstrated decreased *dDA1* expression in the mushroom body. Quantification of dDA1 expression based on image analysis of immunohistochemistry results (Knapek et al., 2011) also confirmed reduced dDA1 expression in the mushroom body (Figure 4B). Administration of a dopamine synthesis inhibitor (tyrosine hydroxylase inhibitor) rescued the short sleep phenotype of *fmn* mutants (Figure 4C) and increased dDA1 expression in the mushroom body (Figures 4D,E). Conditional rescue of *dDAT* in all neurons using the GeneSwitch system (Osterwalder et al., 2001) increased dDA1 expression in the mushroom body (Figure 4F). Without administration of RU486, there was also significant leakage of *dDAT* expression in the mushroom body as previously pointed out (Poirier et al., 2008). To investigate whether the decreased *dDA1* expression was the result of short sleep or increased dopamine signaling, we checked the *dDA1* expression in another sleep mutant. Both calcineurin knockout and knockdown result in the short sleep phenotype (Nakai et al., 2011; Tomita et al., 2011), but *dDA1* expression in the calcineurin mutants was not reduced (Figure 4G). Taken these results together, increased dopamine signaling, rather than short sleep in *fmn* mutants, downregulates *dDA1* expression in the mushroom body. Thus *dDA1* expression level is applicable as an indicator of the dopamine signaling intensity.

### *dDAT* expression rescues downregulation of *dDA1*

**Figure 5:**
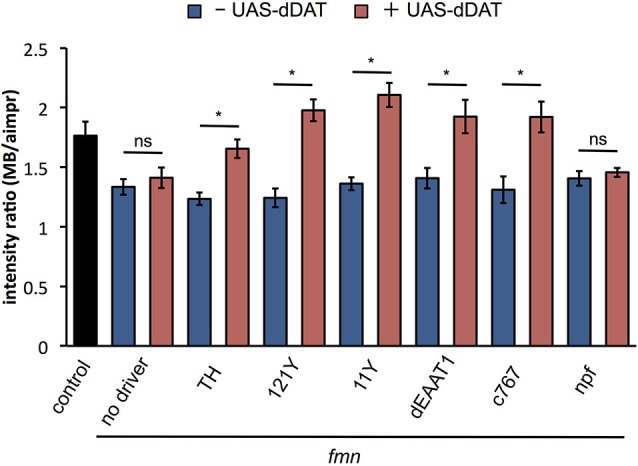
***dDAT* expression rescues *dDA1* down-regulation in the *fmn* mutant**. *dDA1* expression in the mushroom body was calculated as shown in Figure 4B. Error bars represent s.e.m. * *P* < 0.05; two-sided Student’s *t*-test.

To examine the effect of *dDAT* expression on dopamine signaling, we verified the *dDA1* level in *fmn* with *dDAT* expression. As shown in Figure 5, *dDAT* expression in a subset of dopaminergic neurons with TH-GAL4 rescued *dDA1* expression in *fmn*. In addition, *dDAT* expression in the mushroom body (121Y-GAL4, 11Y-GAL4), glial cells (dEAAT1-GAL4) and pars intercerebralis (c767-GAL4) increased *dDA1* expression in the mushroom body, indicating decrease of dopamine signaling. These results are consistent with the rescued sleep phenotype in *fmn* by ectopic *dDAT* expression (Figure 2A). On the other hand, npf-GAL4 driven *dDAT* expression in *fmn* flies could not rescue the short sleep phenotype (Figure 2A) and showed no significant changes in *dDA1* expression in the mushroom body (Figure 5). These results indicate that the ectopic expression of *dDAT* suppresses the increased dopamine signaling in the *fmn* mutant.

### The effect of *dDAT* expression on learning and memory

**Figure 6:**
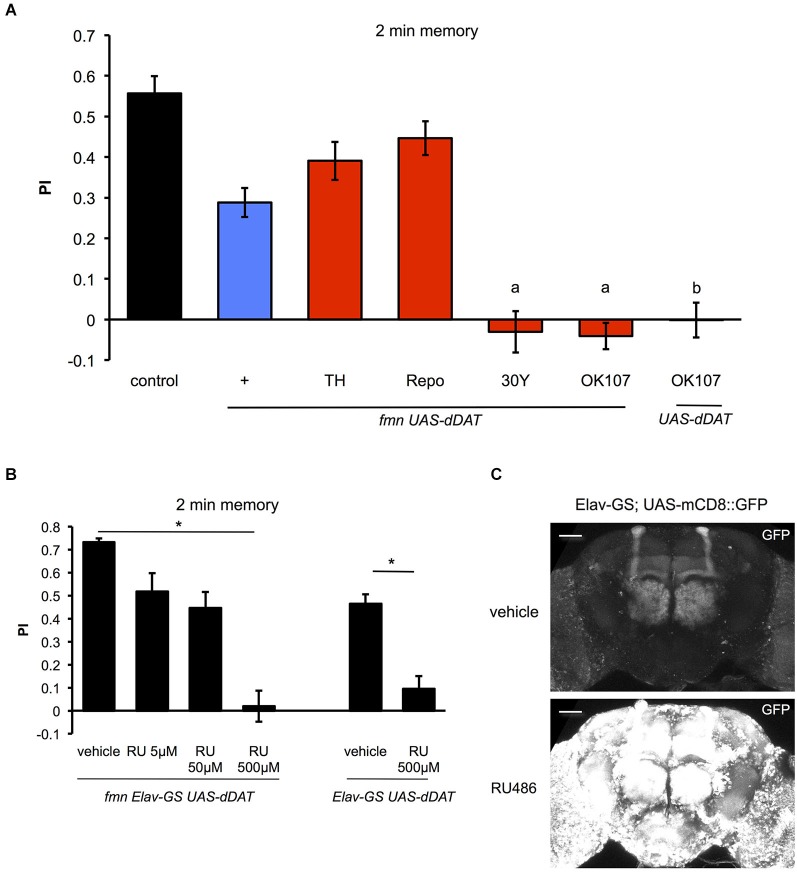
**Effect of *dDAT* expression on memory. (A)** The results of the 2 min memory (immediate memory) tests for each genotype are shown. Each genotype (*n* = 8–17 trials) was tested using an aversive olfactory conditioning test after training. Data are represented as the mean ± s.e.m. a: *P* < 0.05; one-way ANOVA with Tukey-Kramer HSD *post hoc* test. b: *P* < 0.05; two-sided Student’s *t-* test compared with control. **(B)** Conditional expression of *dDAT* in all neurons abolishes memory formation. *fmn Elav-GS UAS-dDAT* or *Elav-GS UAS-dDAT* flies were fed with vehicle or RU486 and then tested using an aversive olfactory conditioning test after training. * *P* < 0.05; one-way ANOVA with Tukey-Kramer HSD *post hoc* test or two-sided Student’s *t-*test. **(C)** Leakage and induction of gene expression with Elav-GS. mCD8::GFP was expressed using the Elav-GS driver. The central brains of flies fed with either vehicle or RU486 are shown. Scale bars = 50 μm.

Next, we investigated the effect of *dDAT* expression on aversive olfactory memory formation. Dopamine signaling mediates associative olfactory memory via *dDA1* receptor in mushroom body (Kim et al., 2007). As previously reported (Zhang et al., 2008), *fmn* mutants have an impaired aversive olfactory memory due to excessive dopaminergic signaling (Figure 6A). *dDAT* expression in glial cells (using Repo-GAL4) as well as dopamine neurons (using TH-GAL4) promoted memory formation in *fmn* mutants although we could not find statistical significance. Surprisingly, however, when some GAL4 drivers which are expressed in the mushroom body (such as 30Y-GAL4 and OK107-GAL4) were used to drive dDAT, these flies did not demonstrate aversive olfactory memory (Figure 6A). 30Y-GAL4 and OK107-GAL4 have broad and strong GAL4 expression in the mushroom body (Aso et al., 2009), which suggest that post-synaptic strong *dDAT* expression abolished dopamine signaling after activation of dopamine neurons with electric punishment. Another possibility is that strong expression of *dDAT* in the mushroom body caused a developmental defect in the Kenyon cells since flies with chemical ablation of mushroom body are unable to perform in a classical conditioning paradigm (de Belle and Heisenberg, 1994). To exclude this possibility, we checked the memory performance of flies with temporal *dDAT* expression using Elav-GeneSwitch. Without administration of RU486, there was significant leakage of *dDAT* expression which may have affected memory performance (Figures 6B,C), since there was a slight improvement in the PI even in flies fed with vehicle (compare control of Figure 6A and vehicle of Figure 6B). However, administration of RU486 results in the impairment of memory performance in a dose dependent manner. Furthermore, the flies fed with 500 μM of RU486 did not demonstrate associative memory (Figure 6B). These results suggest that strong expression of *dDAT* at the post-synaptic site results in abolition of dopamine signaling after activation of dopamine neurons.

## Discussion

In this study, we investigated the effect of *dDAT* expression on sleep and memory. We showed that *dDAT* expression in a subset of dopaminergic neurons is sufficient for normal sleep. We also demonstrated that *dDAT* expression in non-dopaminergic neurons rescue the short sleep phenotype of *fmn* mutant through the reduction of dopaminergic signaling. Finally, we also demonstrated that postsynaptic strong dDAT expression results in the abolishment of memory formation.

To investigate the role of *dDAT* on sleep, we set out to map the neural circuit in which the *dDAT* expression mediates sleep. Mushroom body receives projection of dopaminergic neurons and controls fly sleep (Joiner et al., 2006; Pitman et al., 2006). Previous study demonstrated that *dDA1* expression in mushroom body mediates arousal effect of caffeine and methamphetamine (Andretic et al., 2008). Dopaminergic neurons also have projection to sleep promoting neuropile fan-shaped body and regulates sleep (Donlea et al., 2011; Liu et al., 2012b; Ueno et al., 2012c). Pan-neuronal knockdown of *dDAT* resulted in decrease of sleep similar to *fmn* mutant. On the other hand, flies with *dDAT* RNAi in glial cells or postsynaptic neurons showed no sleep changes which is consistent with the previous report that demonstrated restricted *dDAT* expression in dopaminergic neurons (Porzgen et al., 2001). We also demonstrated that *dDAT* knockdown in a subset of dopaminergic neurons using TH-, HL5-, HL7- and HL9-GAL4 is not sufficient to cause a short sleep phenotype although Ddc-GAL4 driven RNAi resulted in a sleep decrease. TH-GAL4 labels most dopaminergic neurons including fan-shaped body projecting PPL1 and PPM3 clusters but lacks GAL4 expression in PAM clusters. On the other hand, HL5-, HL7- and HL9-GAL4 drivers heavily labels PAM clusters. It is possible that GAL4 expression pattern in dopaminergic neurons is responsible for sleep regulation by *dDAT* since Ddc-GAL4 driven RNAi resulted in a sleep decrease. Another explanation is that *dDAT* expression in serotonin positive neurons contribute to sleep regulation since Ddc-GAL4 labels some serotonin-releasing neurons (Pech et al., 2013). In contrast to the results of knockdown experiments, we demonstrated that *dDAT* expression in a subset of dopaminergic neurons is sufficient to rescue the short sleep phenotype in *fmn* mutant. Taken these results together, we concluded that *dDAT* expression in a subset of dopaminergic neurons is sufficient for normal sleep. It is possible that diverse cells can redundantly fulfill the task of clearing the extracellular space from dopamine since *dDAT* expression in PAM-cluster neurons is not required but sufficient for proper sleep. We further investigated the effect of *dDAT* expression in non-dopaminergic neurons. *dDAT* expression rescued the short sleep phenotype of the *fmn* mutant not only in dopamine neurons but also in other cells such as Kenyon cells and glial cells. The effect of ectopic *dDAT* expression on dopamine signaling was confirmed by the rescue of *dDA1* expression in the mushroom body which is down-regulated in the *fmn* mutant. The ectopic expression of *dDAT* in mushroom body resulted in a poor memory performance. These results may suggest the phenotype of *dDAT* mutant is not only the result of a simple enhancement of physiological dopamine pathway, but also the result of an extrasynaptic volume transmission, i.e., spillover. This should be taken into account when we interpret the phenotype of a neurotransmitter transporter mutant.

Dopamine needs to be cleared from synaptic cleft to maintain the proper signaling levels. dDAT reuptake dopamine from synaptic cleft into presynaptic dopamine neurons. In *Drosophila*, recycling of dopamine through glial cells also regulates dopamine signaling (Suh and Jackson, 2007). Ebony converts dopamine to N-β-alanyl dopamine which is passed from glial cells to presynaptic neurons and then converted back to dopamine. Fly homolog of *Dysbindin*, a human schizophrenia susceptibility gene, regulates dopamine signaling through upregulation of Ebony (Shao et al., 2011). Although we could not find any significant sleep changes with *dDAT* knockdown in glial cell, glial ebony might have contribution to sleep phenotype in fmn with dDAT expression in glial cells. Another mechanism to clear dopamine from synaptic cleft is degradation of dopamine by metabolic enzyme. In *Drosophila*, dopamine is metabolized primarily by arylalkylamine N-acetyltransferase (aaNAT). Flies with aaNAT mutation show defects in sleep homeostasis (Shaw et al., 2000).

Since synaptic and extrasynaptic glutamate uptake transporters are enriched on multiple presynaptic and postsynaptic cells (including glial cells), neurotransmitters such as glutamate are restricted to the synaptic cleft of the release site (Seal and Amara, 1999; Danbolt, 2001). In contrast, neurotransmitters such as gamma-aminobutyric acid (GABA) and monoamines show extrasynaptic (volume) transmission which is mediated by the diffusion of transmitters through the extracellular space (Cragg and Rice, 2004; Syková, 2004; Farrant and Nusser, 2005). Use of experimental methods such as microdialysis or voltammetry with DAT-knockout mice, or psychostimulants such as cocaine or methylphenidate, revealed that DAT inhibition increases the extracellular dopamine concentration (Benoit-Marand et al., 2000; Bradberry et al., 2000), although these invasive methods may affect the extracellular environment. On the other hand, using the fruit fly and its highly sophisticated genetic toolbox we can spatially and temporally control *dDAT* expression and analyze physiological function using behavioral assays. Park *et al*., studied the effect of ectopic *Drosophila* serotonin transporter (*dSERT*) expression in the fly brain and suggested additional factors may be needed for *dSERT* function (Park et al., 2006). By combining ectopic expression, a knockout mutant, a behavioral assay and immunohistochemistry, we revealed that ectopic *dDAT* expression affects the kinetics of dopamine elimination from the synaptic cleft under physiological conditions (i.e., without invasive experimental methods). Cell-type-specific ectopic expression of transporters for neurotransmitters in *Drosophila* will be a favorable tool by which the mechanisms underlying precise inter-cellular signaling in space and time will be elucidated.

A previous study explored the relative roles of the DAT on striatal extracellular dopamine during tonic vs. phasic activity of dopamine neurons in the substantia nigra (Floresco et al., 2003). They suggested that dopamine released during burst firing is normally rapidly removed by uptake before it can escape the synaptic cleft. Although the fly brain is tiny for electrophysiology, ectopic expression of dDAT in the fly brain might shed light on the relationship between physiological function and neural activity of dopamine neurons.

## Conflict of interest statement

The authors declare that the research was conducted in the absence of any commercial or financial relationships that could be construed as a potential conflict of interest.
